# Prediction model of thrombocytopenia on patients using linezolid: a ranked-based covariate selection with multi-center data

**DOI:** 10.3389/fphar.2026.1802397

**Published:** 2026-06-16

**Authors:** Nhi H. Nguyen, Hieu Le, An Q. Tang, Hang T. H. Nguyen, Ha N. Tran, Tuyen T. Nguyen, Thuc T. N. Bui, Anh T. L. Dang, Oanh T. Khuat, Hoa D. Vu, Nhung T. H. Trinh, Anh H. Nguyen

**Affiliations:** 1 National Centre for Drug Information and Adverse Drug Reactions Monitoring, Hanoi University of Pharmacy, Hanoi, Vietnam; 2 Administration of Science Technology and Training, Ministry of Health, Hanoi, Vietnam; 3 Department of Pharmacy, Clinical Pharmacy and Drug Information Unit, Bach Mai Hospital, Hanoi, Vietnam; 4 Department of Pharmacy, Thanh Nhan Hospital, Hanoi, Vietnam; 5 Department of Pharmacy, National Hospital for Tropical Diseases, Hanoi, Vietnam; 6 Clinical Pharmacy and Pharmacoepidemiology Research Group, UiT - The Arctic University of Norway, Tromsø, Norway

**Keywords:** clinical model, linezolid, logistic regression, risk prediction, thrombocytopenia

## Abstract

**Background:**

Thrombocytopenia is a common adverse reaction of linezolid, often leading to severe complications. It is challenging to externally validate directly apply developed in other countries. We aim to develop and validate a risk prediction model of linezolid-associated thrombocytopenia (LAT) tailored to Vietnamese setting and to construct a simplified risk score calculation to support clinical decision-making.

**Materials and Methods:**

Data was collected retrospectively from three large hospitals in Northern Vietnam. We selected inpatients treated with linezolid from November 2019 to March 2023. Potential predictors were chosen based on literature review and clinical experts’ opinions. Final predictors were selected using Bayesian model selection. Thrombocytopenia was defined as platelet count value ≤112.5 G/L and a decrease more than 25% from the baseline. A multivariable logistic regression model was constructed to predict the occurrence of LAT. The final model was further validated using internal-external cross-validation.

**Results:**

Of 776 patients included, 247 patients (31.8%) developed LAT. Logistic regression model selection indicated that the risk predictors were age, duration of linezolid ≥14 days, baseline platelet count, creatinine clearance, sepsis, cirrhosis, continuous renal replacement therapy (CRRT) and heparin use. The model had moderate discrimination, with area under the curve (AUC) of 0.77 (95% confidence interval (CI): 0.72–0.83). Model calibration was good, with calibration-in-the-large and calibration slope of 0.00 (−0.38 to 0.38), and 0.92 (0.59–1.26) respectively. A risk score scale was established, with the optimal cut-off value being 23 points. Patients were categorised based on this score into three groups: low risk (−1 to 13 points), moderate risk (14–22 points) and high risk (≥23 points).

**Conclusion:**

Our newly developed risk prediction model demonstrated moderate discriminatory ability in predicting the occurrence of LAT. From there, a simplified risk score was constructed to facilitate its applicability in clinical practice.

## Introduction

Linezolid is an antibiotic belonging to the oxazolidinone class which selectively inhibits bacterial protein synthesis processes in Gram-positive organisms, exhibiting minimal cross-resistance to other antibiotics. It has antimicrobial spectrum on multi-resistant Gram-positive infections, including *Streptococcus* spp.,. methicillin-resistant *Staphylococcus aureus* (MRSA), and vancomycin-resistant *Enterococcus* (VRE) (Leach et al., 2011). This agent also has favorable pharmacokinetics properties, such as excellent oral bioavailability (∼100%), good penetration and distribution into a variety of tissue, and no need for dose adjustment in renal impairment (Leach et al., 2011). These advantages make linezolid an attractive choice for treating patients with complicated Gram-positive infections. Linezolid is classified among “Reserve” group of the WHO AWaRe classification, which is reserved to treat severe infections caused by multidrug-resistant pathogens (Zanichelli et al., 2023).

A major safety concern of linezolid use is reversible myelosuppression (including anemia, leucopenia, pancytopenia and thrombocytopenia) (The Electronic Medicines Compendium, 2017). The most common adverse reactions of linezolid is thrombocytopenia, with incidence reported in a meta-analysis of 40 observational studies being 37% (Zhang et al., 2023), higher than the 2.4% figure of a phase III clinical study conducted by the manufacturer (The Electronic Medicines Compendium, 2017). Thrombocytopenia can lead to drug discontinuation, prolonged hospital treatment, platelet transfusion, bleeding and mortality (Duan et al., 2022). Therefore, it is crucial to identify patients at higher risk of developing linezolid-associated thrombocytopenia (LAT) and intervene at an early stage.

The risk factors for LAT have been reported in several studies, yet results are inconsistent. A meta-analysis conducted in 2023 has identified some important factors associated with the incidence of LAT including advanced age, body mass index, concurrent renal impairment or liver disease, abnormal laboratory parameters (including white blood cell count, serum creatinine, baseline platelet count, serum albumin, creatinine clearance rate, and estimated glomerular filtration rate), treatment duration and renal replacement therapy (Zhang et al., 2023).

Few studies have established risk prediction models for LAT. However, their sample sizes are quite small (approximately 300–500 patients), and only include patients from a single cent (Duan et al., 2022; Maray et al., 2022; Qin et al., 2021). Using a large dataset with variety of information (e.g., concomitant drugs and comorbidities) collected from multiple centers could be helpful in making the predictions more generalizable.

In this study, aimed to (1) evaluate factors related to LAT and developed a risk prediction model of LAT adapted to Vietnamese setting, (2) perform an internal-external validation to better assess the performance of our model and (3) construct a simplified risk score based on this model to facilitate its applicability in clinical practice (Leach et al., 2011).

## Materials and methods

### Data source

Data was extracted from three hospitals in Northern Vietnam: Bach Mai Hospital is a general hospital with 3,200 beds, National Hospital for Tropical Diseases is a tertiary hospital specialized in infectious diseases, and both are national-level; Thanh Nhan Hospital is a province-level hospital in Hanoi with 900 beds. These hospitals frequently treat severe infections which require the use of linezolid. Data was collected retrospectively from 1 November 2019 to 31 March 2023 from three hospitals, each in two periods. Individual IDs were assigned to each patient’s hospital admission.

### Study population

Patients hospitalized and treated with linezolid during the above-mentioned periods were included. Patients with at least one of the following criteria were excluded: (1): under 18 years of age (2); treated with linezolid for less than 3 days (3); missing recorded platelet count in the period before or after initiation of linezolid therapy (4); having baseline platelet count of >450 G/L; or (5) missing data on the chosen predictors. Each patient was included once per admission and the first linezolid treatment course was evaluated. We followed-up included patients until discharge.

### Outcome

LAT was defined as (1) a platelet count of <112.5 G/L (75% of the lower limit of normal) for patients with a baseline platelet count in the normal range (i.e., 150–450 G/L) or (2) a reduction in platelet count of ≥25% from the baseline value for patients with a baseline platelet count of <150 G/L (The Electronic Medicines Compendium, 2017; Xu et al., 2023; Kawasuji et al., 2021).

Baseline platelet count was measured using the last recorded value before the start of linezolid therapy. LAT could be identified at any time during linezolid treatment or within 5 days after the end of treatment.

### Structured rank-based covariates prioritization

We included the following clinical and demographic characteristics at the start of linezolid treatment for each patient: age, gender, weight, clinical department, comorbidities, laboratory results, and invasive procedures. Regarding drug use characteristics, we evaluated linezolid use data (daily dose per weight, duration, and route of administration) and concomitant medications. Here, we only included concomitant medications having thrombocytopenia as an adverse drug reaction with a frequency of at least >1/1,000 in the information leaflet or Thomson Micromedex DrugDex@ Compendium.

To obtain clinical experts’ opinions on potential risk factors for linezolid-associated thrombocytopenia (LAT), we designed a survey comprising 66 factors, categorized into patient-related and drug-related groups. We included factors that had been identified as LAT risk factors in previous studies or were readily available in medical records at the time of linezolid initiation. For each factor, experts rated their agreement using a 5-point Likert scale (1 = strongly disagree to 5 = strongly agree) in response to the statement: “Does this factor contribute to the risk of thrombocytopenia in patients receiving linezolid?” Some variables were represented in both continuous and binary forms to explore different modeling approaches. Factors for which at least 70% of experts assigned a score of 4 or higher were considered potential predictors for model development (Hasson et al., 2000). The full survey form is provided in the Supporting Information.

### Sample size consideration

The study’s sample size must be sufficient for the purpose of model development. Riley et al. proposed a set of criteria to estimate minimum sample size for models developed using logistic regression, in order to reduce potential of overfitting and ensure the overall risk in the population is estimated precisely (Riley et al., 2019). Using these criteria, we calculated the maximum of predictor parameters that can be screened given the sample size so that the above goals are met.

### Data analysis

We used logistic regression to assess the associations between using linezolid and thrombocytopenia. Variables with very low frequency were excluded prior to regression analysis to avoid unstable estimates due to sparse data. The development of our risk prediction model and risk score followed three steps:

- Model development: Candidate variables were chosen from the clinical experts’ opinion result, with level of agreement >70% (percentage of experts choosing 4 or 5). Expert input was systematically obtained from multiple healthcare professionals in 4 different centres. All variables in the survey were kept as continuous or binary variables (Taylor, 2020). Subsequently, we used Bayesian Model Averaging (BMA) to choose the best predictive model with optimal combination of predictors.
- Model validation: Model was fitted using entire cohort and then evaluated using internal-external validation, which involved splitting the dataset by site (three hospitals) and time periods (Collins et al., 2024a). Five clusters were used in model development and the held-out one was used for validation. These steps were repeated six times, each time taking out a different cluster to examine the generalizability and heterogeneity of the performance (Collins et al., 2024a).
- Risk score construction: A simplified risk score was constructed based on the hierarchy of the regression coefficients in the final model (each coefficient was divided by the smallest coefficient and rounded to the nearest integer). For continuous risk factors, the categories were designed to mirror clinically meaningful risk factor states (Sullivan et al., 2004). Risk of LAT corresponding to each score was calculated.
- Decision curve analysis: Clinical utility was evaluated using decision curve analysis to assess net benefit across a range of threshold probabilities.

Missing data were handled using a complete-case analysis approach. Cases with missing values in any of the variables included in the analysis were excluded, resulting in the exclusion of 176 cases. Missingness primarily occurred in variables related to concomitant medications and invasive procedures.

Data processing and cleaning, regression modelling, evaluation of internal-external validation and constructing risk score scale were performed in R (version 4.4.0). Our reporting adhered to the TRIPOD + AI checklist for reporting clinical prediction models that use regression or machine learning methods (Collins et al., 2024b).

## Results

### Patients characteristics

**FIGURE 1:**
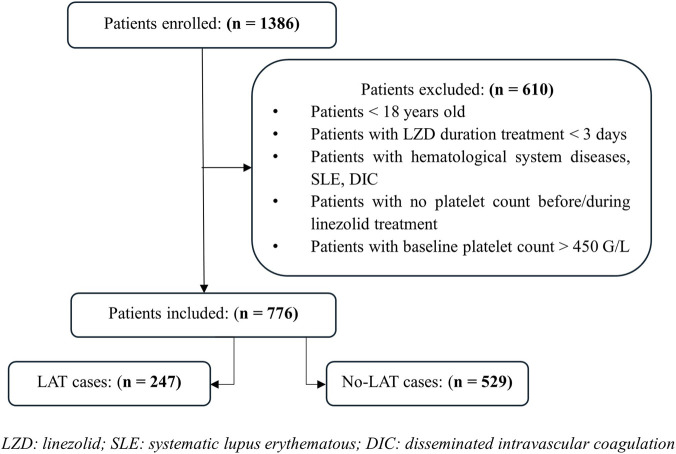
Flowchart of patients included in this study.

Of 1,386 patients administered linezolid during study periods in three hospitals, a total of 776 patients were included in our study (Figure 1). All patients received 600 mg linezolid q12 h. Their median age was 62.0 [50.0–73.0] years, with male accounting for 62.9% (488/776) of the total cohort. LAT occurred in 31.8% (n = 247) of the patients, and it appeared after 5.0 [3.0–10.5] days of linezolid administration. The patient characteristics are shown in Table 1. To minimize instability in regression estimates due to sparse data and potential quasi-complete separation, variables with very low frequency (fewer than three events) were excluded after descriptive analysis and prior to univariable logistic regression.

**TABLE 1:** Patient and drug use characteristics.

​	​	Thrombocytopenia status		Univariate regression		
Characteristic	Overall N = 776^a^	No LAT event N = 529^a^	LAT event N = 247^a^	OR^b^	95% CI^b^	p-value
Demographics						
Age (10 years)	6.2 (5.0–7.3)	6.1 (4.8–7.2)	6.4 (5.5–7.4)	1.18	1.08, 1.30	**<0.001**
Female sex	288 (37%)	195 (37%)	93 (38%)	1.03	0.76, 1.41	0.8
Weight (10 kg)	5.5 (5.0–6.0)	5.5 (5.0–6.0)	5.5 (4.8–6.2)	1.02	0.87, 1.20	0.8
Clinical department						
ICU	513 (66%)	326 (62%)	187 (76%)	1.94	1.39, 2.74	**<0.001**
Other	263 (34%)	203 (38%)	60 (24%)	0.52	0.36, 0.72	**<0.001**
Comorbidities						
Hypertension	330 (43%)	219 (41%)	111 (45%)	1.16	0.85, 1.57	0.4
Diabetes	212 (27%)	145 (27%)	67 (27%)	0.99	0.70, 1.38	>0.9
Heart failure	219 (28%)	126 (24%)	93 (38%)	1.93	1.39, 2.68	**<0.001**
Angina	35 (4.5%)	20 (3.8%)	15 (6.1%)	1.65	0.81, 3.26	0.2
Cirrhosis	47 (6.1%)	19 (3.6%)	28 (11%)	3.43	1.89, 6.37	**<0.001**
COPD	39 (5.0%)	25 (4.7%)	14 (5.7%)	1.21	0.60, 2.34	0.6
Cerebral vascular accident	88 (11%)	63 (12%)	25 (10%)	0.83	0.50, 1.34	0.5
Myocardial infarction	20 (2.6%)	15 (2.8%)	5 (2.0%)	0.71	0.23, 1.85	0.5
Malignancies	58 (7.5%)	36 (6.8%)	22 (8.9%)	1.34	0.76, 2.31	0.3
Invasive procedures						
Endotracheal intubation	366 (47%)	218 (41%)	148 (60%)	2.13	1.57, 2.91	**<0.001**
Central venous catheter	390 (50%)	225 (43%)	165 (67%)	2.72	1.99, 3.74	**<0.001**
Intermittent hemodialysis	97 (13%)	57 (11%)	40 (16%)	1.60	1.03, 2.47	**0.035**
Continuous renal replacement therapy	130 (17%)	60 (11%)	70 (28%)	3.09	2.10, 4.56	**<0.001**
Laboratory tests						
WBC (10 G/L)	1.2 (0.82–1.7)	1.2 (0.83–1.7)	1.2 (0.78–1.8)	1.09	0.90, 1.31	0.4
HGB (10 G/L)	10 (9.0–12)	11 (9.2–12)	9.8 (8.6–12)	0.89	0.83, 0.95	**<0.001**
PLT (10 G/L)	21 (15–29)	24 (17–31)	16 (11–21)	0.92	0.90, 0.94	**<0.001**
CLCR (10 mL/min)	4.9 (2.1–8.3)	5.5 (2.6–8.8)	3.3 (1.6–6.4)	0.90	0.86, 0.93	**<0.001**
Kidney function						
CLCR <30	272 (35%)	153 (29%)	119 (48%)	4.07	2.35, 7.42	**<0.001**
30 ≤ CLCR <60	190 (24%)	127 (24%)	63 (26%)	2.60	1.45, 4.85	**0.002**
60 ≤ CLCR <90	157 (20%)	119 (22%)	38 (15%)	1.67	0.90, 3.22	0.11
90 ≤ CLCR <130	106 (14%)	89 (17%)	17 (6.9%)	—	—	​
CLCR ≥ 130	51 (7.0%)	41 (8%)	10 (4.1%)	1.28	0.52, 2.99	0.6
Existing infections						
Hospital-acquired pneumonia	353 (45%)	243 (46%)	110 (45%)	0.95	0.70, 1.28	0.7
Septicemia	240 (31%)	161 (30%)	79 (32%)	1.07	0.77, 1.49	0.7
Skin and soft tissue infection	139 (18%)	101 (19%)	38 (15%)	0.77	0.51, 1.15	0.2
Sepsis	121 (16%)	53 (10%)	68 (28%)	3.41	2.30, 5.10	**<0.001**
Community-acquired pneumonia	116 (15%)	68 (13%)	48 (19%)	1.64	1.09, 2.45	**0.017**
Central nervous system infection	62 (8.0%)	44 (8.3%)	18 (7.3%)	0.87	0.48, 1.51	0.6
Urinary tract infection	48 (6.2%)	32 (6.0%)	16 (6.5%)	1.08	0.57, 1.97	0.8
Intra-abdominal infection	47 (6.1%)	33 (6.2%)	14 (5.7%)	0.90	0.46, 1.69	0.8
Bone and joint infection	12 (1.5%)	11 (2.1%)	1 (0.4%)	0.19	0.01, 0.99	0.11
Linezolid use						
Dose (mg/kg)	22 (20–24)	22 (20–24)	22 (19–25)	1.00	0.96, 1.04	0.9
Duration (days)	9.0 (6.0–14)	9.0 (6.0–13)	10 (6.0–14)	1.03	1.00, 1.06	**0.020**
IV route	756 (97%)	513 (97%)	243 (98%)	1.89	0.69, 6.67	0.3
Comedications						
Carbapenem	565 (73%)	371 (70%)	194 (79%)	1.56	1.10, 2.24	**0.015**
Levofloxacin	240 (31%)	156 (29%)	84 (34%)	1.23	0.89, 1.70	0.2
Teicoplanin	38 (4.9%)	23 (4.3%)	15 (6.1%)	1.42	0.71, 2.75	0.3
Daptomycin	1 (0.1%)	1 (0.2%)	0 (0%)	​	​	​
Ibuprofen	19 (2.4%)	14 (2.6%)	5 (2.0%)	0.76	0.24, 2.01	0.6
Tacrolimus	1 (0.1%)	0 (0%)	1 (0.4%)	​	​	​
Valproic acid	32 (4.1%)	22 (4.2%)	10 (4.0%)	0.97	0.43, 2.03	>0.9
Carbamazepine	8 (1.0%)	8 (1.5%)	0 (0%)	0.00	​	>0.9
Quetiapine	4 (0.5%)	4 (0.8%)	0 (0%)	0.00	​	>0.9
Enoxaparin	358 (46%)	235 (44%)	123 (50%)	1.24	0.92, 1.68	0.2
Heparin	215 (28%)	106 (20%)	109 (44%)	3.15	2.27, 4.39	**<0.001**
Clopidogrel	40 (5.2%)	30 (5.7%)	10 (4.0%)	0.70	0.32, 1.41	0.3
Rifampin	15 (1.9%)	7 (1.3%)	8 (3.2%)	2.50	0.89, 7.20	0.081
Pyrazinamide	13 (1.7%)	5 (0.9%)	8 (3.2%)	3.51	1.16, 11.7	**0.029**

a Median (Q1 - Q3); n (%)

b OR = Odds Ratio, CI = Confidence Interval

Bold text in the Characteristic column indicates category headings; bold p-values indicate statistical significance (p < 0.05).

### Potential predictors

**FIGURE 2:**
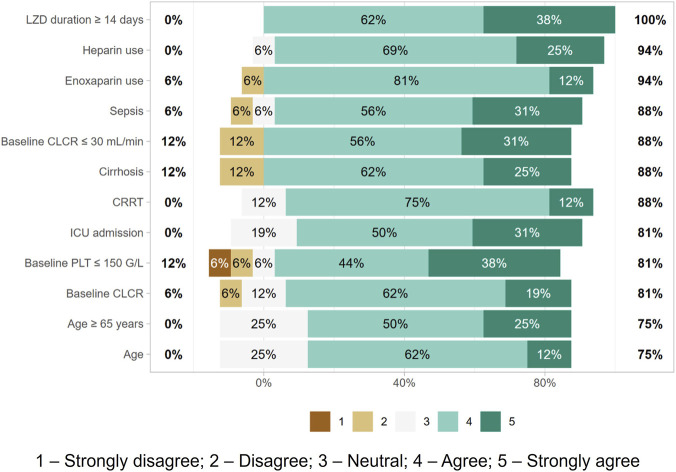
Clinical expert’s opinion survey results on risk factors for linezolid-associated thrombocytopenia (LAT).

A total of 11 clinical experts participated in the survey (06 physicians and 05 pharmacists). The results identified 12 potential predictors: age (continuous and binary with a cut-off at 65 years), baseline creatinine clearance (continuous and binary with a cut-off at 30 mL/min), baseline platelet count (continuous), linezolid treatment duration (binary, with a cut-off at 14 days), ICU admission, continuous renal replacement therapy, septic shock, cirrhosis, heparin use, and enoxaparin use. The detailed results, including the perceived importance of each predictor as rated by clinical experts on a 5-point Likert scale, are presented in Figure 2. The assumption of linearity between continuous predictors and the log-odds of the outcome was assessed using exploratory plots. As no substantial non-linear relationships were observed, continuous variables were retained without transformation, including age, baseline creatinine clearance, and baseline platelet count.

### Model development

The results of multivariable logistic regression analysis are presented in Table 2. Multi-collinearity among variables selected in the final model was low (VIFs 1.05–1.29). Final model contained eight predictors: age, creatinine clearance (ClCr), baseline platelet count (PLT), cirrhosis, septic shock, continuous renal replacement therapy (CRRT), linezolid duration and heparin use.

**TABLE 2:** Predictors of linezolid-associated thrombocytopenia included in final model.

Coefficient	Estimate	OR (95% CI)	p-value
Intercept	−0.731	​	0.145
Age (per 10 years)	0.181	1.20 (1.07–1.35)	0.003
Creatinine clearance (per 10 mL/min)	−0.053	0.95 (0.90–1.00)	0.041
Baseline platelet count (10 G/L)	−0.081	0.92 (0.90–0.94)	<0.001
Cirrhosis	0.824	2.28 (1.15–4.60)	0.019
Septic shock	0.594	1.81 (1.14–2.88)	0.012
Continuous renal replacement therapy	0.410	1.51 (0.94–2.42)	0.090
Linezolid duration ≥14 days	0.986	2.68 (1.80–4.01)	<0.001
Heparin use	0.878	2.41 (1.61–3.60)	<0.001

Based on the regression coefficients and intercept, a risk prediction model for LAT was established as below (events are coded as “1” if present and “0” if absent):

Probability of thrombocytopenia = 1/[1+ exp-(-0.731 + 0.986 × linezolid duration ≥14days +0.878 × Heparin use +0.594 × Septic shock – 0.053 × ClCr +0.181 × Age – 0.081 × PLT +0.824 × Cirrhosis +0.410 × CRRT)].

### Model validation

The model showed good discrimination, ranging between AUROC index of 0.71 (95% CI 0.59–0.80) in Thanh Nhan phase 1 to 0.84 (95% CI 0.73–0.91) in Bach Mai phase 2. A pooled random effects meta-analysis estimate is 0.77 (95% CI 0.72–0.83).

**FIGURE 3:**
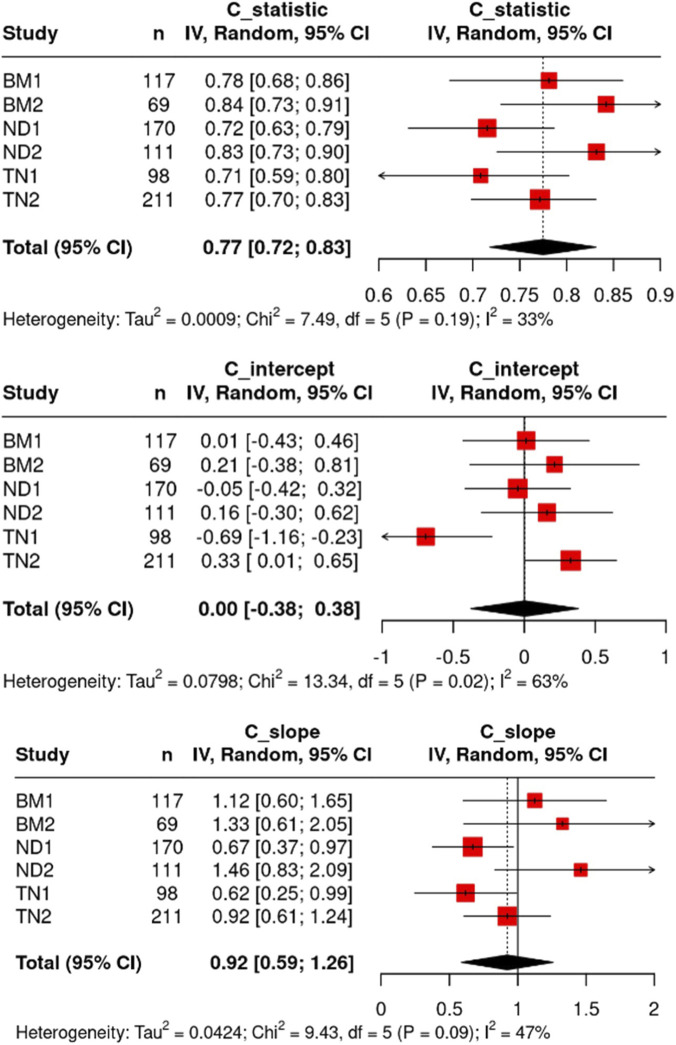
Discrimination and calibration parameters for internal-external validation.

Pooled calibration metrics indicated mild systemic miscalibration. The meta-analysis pooled calibration slope was 0.92 (95% CI 0.59–1.26), and calibration-in-the-large (calibration intercept) of the model is 0.00 (95% CI -0.38 to 0.38). Both calibration metrics showed moderate heterogeneity. Discrimination (AUROC or c-statistic) and calibration (intercept and slope) metrics from the internal–external validation are summarized in Figure 3.

### Risk score construction

The risk score was based on the hierarchy of the corresponding regression coefficients, with 10 years as the reference coefficient. Baseline platelet count was categorized according to the Common Terminology Criteria for Adverse Events (CTCAE) grading scale for thrombocytopenia. Full risk score was presented in Table 3.

**TABLE 3:** Risk score scale for developing thrombocytopenia.

Risk factors	Score	Risk factors	Score
**Age**	​	**Baseline platelet count (G/L)**	​
18–29	0	≥150	0
30–39	1	75 ≤ PLT <150	8
40–49	2	50 ≤ PLT <75	10
50–59	3	25 ≤ PLT <50	11
60–69	4	<25	12
70–79	5	**Septic shock**	​
80–89	6	No	0
≥90	7	Yes	3
**Creatinine clearance (mL/min)**	​	**CRRT**	​
≥130	−1	No	0
90 ≤ ClCr <130	0	Yes	2
60 ≤ ClCr <90	1	**LZD duration ≥ 14 days**	​
30 ≤ ClCr <60	2	No	0
<30	3	Yes	5
**Cirrhosis**	​	**Heparin use**	​
No	0	No	0
Yes	5	Yes	5

PLT: platelet count; CRRT: continuous renal replacement therapy; LZD: linezolid. Bold values are names of risk factors.

The score has a range from −1 to 42 points, with corresponding probabilities of LAT to from 3.2% to 98.7%. Age from 18 to 29 corresponds to 0 points, and for every 10-year increase in age, there will be an increase of 1 point. Creatinine clearance between 90 and less than 130 mL/min corresponds to 0 points. For every decrease in ClCr of 30 mL/min, the risk score increases by 1 point. Initial platelet count ≥150 G/L counts as 0 points, initial PLT <25 G/L has the greatest impact with 12 points.

The optimal cut-off point was determined using the Youden index (J). Based on this approach, a threshold of 23 points was selected, corresponding to a predicted probability of thrombocytopenia of 71.2%. We categorized patients based on this score into three groups: low risk (−1 to 13 points), moderate risk (14–22 points) and high risk (≥23 points).

### Decision curve analysis

Decision curve analysis demonstrated that the model provided a higher net benefit compared to both “treat all” and “treat none” strategies across a range of threshold probabilities (approximately 10%–70%) (Supplementary Figure S4). Within this range, the model consistently showed superior clinical utility. At lower and higher threshold probabilities, the net benefit of the model approached that of the default strategies.

## Discussion

In this study, we developed and validated a risk prediction model to predict LAT in Vietnamese setting. The model comprised of eight predictors: age, creatinine clearance, baseline platelet count, cirrhosis, septic shock, continuous renal replacement therapy, linezolid duration and heparin use. A simplified risk score was constructed to facilitate its applicability of the prediction rule in practice.

We collected data for inpatients administered with linezolid at a fixed dose of 600 mg q12 h in three hospitals (Bach Mai hospital, Thanh Nhan hospital and National Hospital for Tropical Diseases). These hospitals were chosen because of existing antimicrobial stewardship programs for linezolid, with studies investigating drug use evaluation (DUE) for linezolid. Hence, we accumulated quite a complete set of data on patient characteristics, drug use characteristics, as well as adverse events including thrombocytopenia.

Previous studies developing logistic regression models for LI-TP risk predictions have included 4-6 predictors in their final models (Duan et al., 2022; Xu et al., 2023; Liu et al., 2023). We expected to include about as many candidate predictors, based on results from the expert opinion survey and the Bayesian Model Selection algorithm. Some of the candidate predictors might be continuous, which may potentially require non-linear modelling and therefore slightly increase the number of parameters. Before model development, we find it acceptable to include continuous predictors as linear terms only, therefore each predictor only uses one parameter. Prior to model development, we assessed the functional form of continuous predictors by examining their relationship with the log-odds of the outcome. Based on these exploratory analyses, the relationships for age, baseline creatinine clearance, and baseline platelet count were considered approximately linear, and no strong evidence of non-linearity was observed. With a sample size of 776, an estimated LAT prevalence of 37%, and a conservative Nagelkerke’s *R* ^2^ of 0.15, we estimated that we can screen at most 21 predictor parameters. Our model was developed after screening only 12 parameters, therefore our sample size is entirely sufficient to develop this model.

The incidence of LAT recorded in our study is 31.8%, which was quite high but consistent with those from previous studies, ranging from 16.7% to 64.7% (Niwa et al., 2009; Nukui et al., 2013; Chen et al., 2012). Xu *et al.* reported lower incidence, with 28.5% patients experienced LAT (Xu et al., 2023). The reason could be the difference in exclusion criteria, as their study excluded patients with high probability of developing thrombocytopenia such as having baseline platelet count <75 G/L, severe liver impairment (Child-Pugh C), under hemodialysis and using antiplatelet drugs.

In our study, LAT appeared after 5.0 [3.0–10.5] days of linezolid administration. We found that the duration of linezolid treatment was a risk factor for LAT, and chose the cut-off value based on previous studies (Maray et al., 2022; Takahashi et al., 2021; Hirano et al., 2014). The lack of a linear correlation between this factor and thrombocytopenia events led to its classification as a binary variable. This simplification also facilitates clinical utilization of the risk score during linezolid prescription.

We found that a 10-year increase in age corresponds to a 1.20-fold odd of LAT, which may be associated with slow linezolid metabolism or poor bone marrow hematopoietic function as patients get older. Liu et al. (2023) also included age in their final model using cut-off at 80 years (OR 2.04, p < 0.001) (Liu et al., 2023). Tinelli *et al.* observed that serum trough concentration of linezolid surpassed the normal threshold (8.0 mg/L) in elderly patients following administration of the standard dose (600 mg twice daily) (Tinelli et al., 2017). Consequently, the risk of LAT escalates with advancing age.

Several studies have shown a significant association between renal function impairment and LAT, and high trough concentration of linezolid is an independent risk factor (Dai et al., 2021; Dong et al., 2014). Pharmacokinetic studies of linezolid have indicated that 30% of linezolid is eliminated unchanged via the kidneys and the clearance rate of linezolid decreases by 20% in renal failure (The Electronic Medicines Compendium, 2017), which leads to an increase in the blood concentration of linezolid, thereby inducing LAT. Takahashi *et al.* found that when creatinine clearance was <50 mL/min, the odd of LAT was 2.32 times, and LAT occurred earlier (6.7 ± 4.4 days vs. 8.5 ± 5.2 days, P = 0.039) (Takahashi et al., 2021). In our investigation, we observed an inverse relationship between the risk of thrombocytopenia and creatinine clearance. Unlike previous studies that frequently dichotomize creatinine clearance using a threshold value to create a binary variable, our findings decided creatinine clearance as a continuous variable.

A low baseline platelet count is a risk factor for LAT, which has been demonstrated in many studies, with threshold ranging from 90–240 G/L (Xu et al., 2023; Choi et al., 2019). Our study reported the difference in baseline platelet count between patients developing thrombocytopenia and those did not (median was 157 and 239 G/L respectively). The baseline platelet count in our study exhibited a linear association with the risk of thrombocytopenia, hence we included as a continuous variable. Since the criteria for defining LAT is a decrease in platelet count of ≥150 × 10 G/L or a 25% reduction, it is obvious that for lower baseline counts, a smaller decrease is required to meet the criteria for LAT. Therefore, the association between low platelet baseline count and higher risk for LAT is expected.

Heparin appeared in the final model as a significant risk factor (OR 2.41; 95% CI 1.61–3.60). It is possible that concomitant unfractioned heparin use is a risk factor for LAT. Conversely, this finding may be related to other factors. We cannot exclude the possibility that patients experienced heparin-induced thrombocytopenia. Usually, unfractioned heparin is used for prophylaxis of venous thromboembolism in patients with renal impairment. The higher frequency of thrombocytopenia among patients receiving concomitant unfractioned heparin may relate to these patients having renal impairment, which would place them at higher risk of LAT.

Continuous renal replacement therapy was not a significant predictor in our findings (OR 1.51, 95% CI 0.94–2.42, p = 0.090). However, as the goal of our study is to develop a performant and useful model rather than to identify independent causal factors, predictors were selected based on their contribution to overall model performance rather than individual statistical significance. Accordingly, this variable was retained as part of the final model.

Interestingly, septic shock is a predictor included in our final model. Other studies did not report septic shock as an independent risk factor. Furthermore, thrombocytopenia is frequently observed in sepsis and septic shock. Its etiology involves multiple mechanisms, such as underlying bone marrow dysfunction, impaired megakaryopoiesis, robust activation of intravascular coagulation pathways, microvascular coagulopathy, blood loss, and heightened peripheral platelet consumption.

We found that patients with cirrhosis had a 2.28-fold odd of LAT, the reason could be cirrhosis significantly reduces linezolid clearance. Hepatic pathophysiological alterations during progressive fibrosis involve the downregulation of proteins associated with hepatic oxidative reactions, impacting linezolid metabolism. The fibrotic process disrupts liver lobule structures, resulting in diminished drug and oxygen transport to liver cells, consequently leading to reduced linezolid clearance. Ikuta et al. (2011) identified chronic liver disease as an independent risk factor; and other studies included different indicators of liver function such as albumin or bilirubin (Liu et al., 2023).

Clinicians can use the risk score to determine a patient’s risk of LAT and thus making suitable interventions. According to LAT incidence, patients were classified as low risk, moderate risk and high risk. In low risk group, patient monitoring of blood routine may be not required, or at least once per week as recommendation of manufacturer (The Electronic Medicines Compendium, 2017). In moderate risk group, we can monitor platelets count more closely and stop linezolid as soon as possible; for high risk group, clinicians should consider carefully about the risk-benefit balance before prescribing linezolid.

For ease of use in clinical practice, the final model was transformed into a simplified risk score, allowing straightforward risk stratification without requiring complex calculations. A cut-off of 23 points was selected to distinguish high-risk patients using the Youden (J) index. Notably, most patients in our study were classified as low- or moderate-risk, corresponding to threshold probabilities within the range where the model demonstrated the greatest net benefit in decision curve analysis (approximately 10%–70%). This suggests that the model may be particularly useful in supporting clinical decision-making in patients with uncertain risk. In contrast, for patients classified as high risk (≥23 points), the net benefit of the model approached that of the “treat none” strategy, indicating limited incremental value of model-based decision-making in this subgroup, where clinical decisions may be more straightforward.

### Strengths and limitations

To the best of our knowledge, the present study represents the first investigation into the predictor for with LAT using a multi-center database in Vietnam. Our model development methodology considers a range of factors, including patient characteristics and drug usage patterns. Rather than relying solely on statistical analysis, we integrated clinical experts’ opinions to identify the most significant and clinically relevant variables. Although expert-informed pre-selection may introduce subjectivity, we minimized this risk by using a structured approach with input from multiple clinicians. Our objective of this study is to ensure that the prediction model includes clinically meaningful variables and aligns with real-world practice, rather than relying solely on data-driven methods. Furthermore, clinical utility was evaluated using decision curve analysis, which assesses the net benefit of the model across a range of threshold probabilities.

However, there are some limitations in this study that need to be taken into account. First, this was a retrospective study, so we were unable to confirm the accuracy of the data and to ascertain the missing data. Missing data were handled using a complete-case analysis approach, which may introduce selection bias and potentially affect model estimates. Although more advanced methods such as multiple imputation could be considered, we opted for a complete-case approach given the nature and distribution of missingness, to maintain transparency and avoid introducing additional assumptions. Second, baseline platelet count was included as a predictor despite being related to the outcome definition, which may introduce partial circularity and potentially inflate model performance. The causal relationship between linezolid exposure and thrombocytopenia could not be evaluated; thus, we could not ascertain whether other diseases and/or other drugs affect platelet count. Furthermore, we could not monitor blood concentration of linezolid since the procedure had not been applied in any hospital included hence, the possibility that high linezolid blood concentration being a confounding factor cannot be eliminated. Finally, model performance was assessed using an internal-external validation approach across subsets of the multicenter dataset to evaluate robustness and generalizability. Nevertheless, further validation in a completely independent clinical setting is required to confirm model transportability. An external validation study in another hospital is currently underway to further evaluate the model’s clinical applicability.

## Conclusion

In summary, the incidence of LAT was approximately 30% in our study which comprises of Vietnamese patients. Platelet counts should be closely monitored in patients who use linezolid for more than 14 days, those with higher age, renal impairment, cirrhosis, CRRT, lower baseline platelet count, septic shock experience, and concurrent heparin use. A logistic regression model based on these predictors demonstrated moderate discrimination. Establishing a risk score could assist clinicians in conveniently stratifying patients into different risk groups for developing thrombocytopenia while receiving linezolid therapy and implementing appropriate measures. Further external validation is warranted to confirm its performance in independent populations.

## Data Availability

The data that support the findings of this study are available on request from the corresponding author. The data are not publicly available due to privacy or ethical restrictions. Requests to access these datasets should be directed to Anh H. Nguyen, anh90tkvn@gmail.com.

## Supplementary material

SUPPLEMENTARY INFORMATION S1 Clinical expert’s opinion survey form.

SUPPLEMENTARY FIGURE S1 Linearity check–Assessment of linearity between continuous predictors and the log-odds of LAT.

SUPPLEMENTARY FIGURE S2 Relationship between predictors and LAT.

SUPPLEMENTARY FIGURE S3 Calibration plots.

SUPPLEMENTARY FIGURE S4 Decision curve analysis of the prediction model.

SUPPLEMENTARY TABLE S1 The predictive capability at each score level.

SUPPLEMENTARY TABLE S2 Conversion table from regression coefficients to risk integer score.

SUPPLEMENTARY TABLE S3 TRIPOD + AI checklist for reporting clinical prediction models that use regression or machine learning methods.
